# Spectral boundary integral method for simulating static and dynamic fields from a fault rupture in a poroelastodynamic solid

**DOI:** 10.1007/s40948-022-00368-4

**Published:** 2022-03-25

**Authors:** Elías Rafn Heimisson, Antonio Pio Rinaldi

**Affiliations:** ETH Zürich, Sonneggstrasse 5, 8092 Zürich, Switzerland

**Keywords:** Earthquakes, Induced seismicity, Poroelastodynamics, Boundary integral method, Waveform simulations

## Abstract

The spectral boundary integral method is popular for simulating fault, fracture, and frictional processes at a planar interface. However, the method is less commonly used to simulate off-fault dynamic fields. Here we develop a spectral boundary integral method for poroelastodynamic solid. The method has two steps: first, a numerical approximation of a convolution kernel and second, an efficient temporal convolution of slip speed and the appropriate kernel. The first step is computationally expensive but easily parallelizable and scalable such that the computational time is mostly restricted by computational resources. The kernel is independent of the slip history such that the same kernel can be used to explore a wide range of slip scenarios. We apply the method by exploring the short-time dynamic and static responses: first, with a simple source at intermediate and far-field distances and second, with a complex near-field source. We check if similar results can be attained with dynamic elasticity and undrained pore-pressure response and conclude that such an approach works well in the near-field but not necessarily at an intermediate and far-field distance. We analyze the dynamic pore-pressure response and find that the P-wave arrival carries a significant pore pressure peak that may be observed in high sampling rate pore-pressure measurements. We conclude that a spectral boundary integral method may offer a viable alternative to other approaches where the bulk is discretized, providing a better understanding of the near-field dynamics of the bulk in response to finite fault ruptures.

**Article highlights**

- We present a spectral boundary integral approach to friction and fracture problems in a poroelastodynamic solid.
- Convolution kernels are constructed with a parallel numerical inversion to Laplace transforms.
- Numerical simulations show that P-wave arrival may carry a significant pore pressure peak.

## Introduction

The spectral boundary integral method (SBIM) in frictional and fracture mechanics is based on the idea of deriving analytical or semi-analytical solutions for an arbitrary Fourier mode in the fracture or interface conditions, for example, the slip. The arbitrary boundary conditions are then obtained by superposition, in other words, representing the slip or other imposed interface conditions as a Fourier series in space at any given time. The main benefit of the approach is that one can utilize the efficiency and desirable scaling properties of the Fast Fourier Transform (FFT) algorithms to compute the Fourier coefficients. Thus, practically speaking, the method avoids explicitly carrying out a computationally expensive spatial convolution that may be needed when implementing fundamental or dislocation solutions for similar purposes. However, there are some notable limitations of the spectral method: first, the approach is mostly limited planar faults or interfaces (with some exceptions: Romanet and Ozawa 2021). Second, the method imposes periodic boundary conditions on the spatial domain (see some discussion in Sect. 4.1 on how this assumption can be removed).

The SBIM has been applied widely to analyze interface frictional and fracture problems both for fully elastodynamic solid and quasi-static elasticity. Some approaches use the FFT algorithm to carry out an efficient spatial convolution of an analytical elastic integral kernel and slip or slip speed (e.g. Quin and Das 1989; Rice 1993). But generally, the SBIM refers to when the analytical solutions are derived directly in the time and wavenumber domains; thus, the convolution kernels in the spatial domain are not needed. This approach has been shown to be particularly efficient for elastodynamic problems (e.g. Perrin and Rice 1994; Geubelle and Rice 1995), where relationships between slip or slip speed and stress on the fracture or fault interface are obtained as convolution kernels in time, but no convolution is needed in space since the convolution kernels are represented in the wavenumber domain. By virtue of the Fourier decomposition and linearity, the operations on each Fourier coefficient are independent of operations of other Fourier coefficients at the same time-step. This modal independence lends itself to a straightforward parallelization of simulations. This property has been particularly useful in fully dynamic simulations on rate-and-state faults, which are particularly computationally expensive due to very large differences in relevant time scales that need to be resolved (Lapusta et al. 2000; Lapusta and Liu 2009). The SBIM implementation for elastodynamics or quasi-static elasticity has generally derived slip to stress relationship on the fault and thus are unable to directly compute off fault fields. A recent exception is the work of Barbot (2021) where the spectral boundary integral approach was extended to multiple parallel faults.

An SBIM for poroelastodynamics has not been presented to date in the same manner as for elastodynamics or quasi-static elasticity. However, fundamental solutions have been derived, Cheng et al. (1991); Dominguez (1992) presented a boundary integral solution in the frequency domain, that is for time-harmonic changes. Time-domain fundamental solutions for points sources were later derived (Chen 1994; Gatmiri and Kamalian 2002). However, in application to earthquake dynamics such fundamental solution may not honour possible non-trivial boundary conditions on the interface pore pressure (Heimisson et al. 2019, 2021). It is, therefore, important to be able to readily alter such boundary conditions.

Here we present a spectral boundary integral approach for fracture, frictional, and faulting problems in a poroelastodynamic solid. In this study, we limit the scope to simply imposing the slip history and analyzing the off fault fields. However, the method, broadly speaking, could be applied to on-fault fields similar to what was done by Lapusta et al. (2000) where the slip history is simulated from a physics-based friction law. We use a numerical inversion of the Laplace transform to obtain convolution kernels in the time and wavenumber domain. The mathematics is carried out directly from the governing differential equations with a symbolic manipulator, and thus imposing changes in boundary conditions and deriving new kernels is typically simple.

With the large number of in-situ experiments currently being performed at various underground laboratories (e.g. Guglielmi et al. 2020, 2021; Ma et al. 2020; Schoenball et al. 2020), it is important to understand which processes may be relevant in the near field of a stimulated fault/fracture. The development of new high-frequency sensors will allow for more detailed measurements of dynamic processes. We suggest that the methods may be used to efficiently analyze such signals in this new era of field experiments in geomechanics and seismology.

This paper first discusses the problem setup (Sect. 1.1), then generally presents the theory (Sect. 2), which includes a discussion of governing equations, boundary conditions, spectral solution strategy, and numerical implementation. In Sect. 3 we present the results, with a focus on the dynamic poroelastic response and we make a comparison to a comparable elastodynamic solution. Finally, some more detailed discussion is offered in Sect. 4.

### Problem setup

**Fig. 1:**
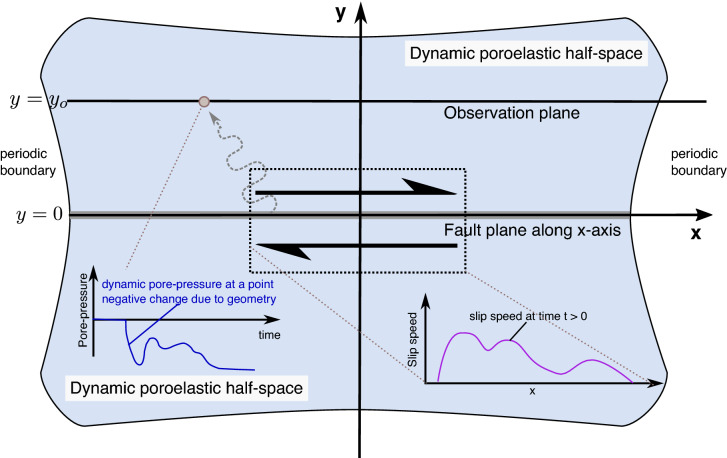
Schematic setup of the problem and simulations. Two identical and isotropic dynamic poroelastic half-spaces (poroelastodynamic) share an interface at $y=0$. The fault, where slip occurs, lies on the *x* axis, while all fields are invariant along the *z*-axis (plane strain, not shown). In the study, we observe the response at a plane $y = y_o$, due to imposed slip at $y=0$. The imposed slip can have arbitrary spatial and temporal behaviour as long as it is well resolved by the discretization. At the observation plane, we can construct any relevant field, for example, the dynamic pore-pressure response due to the imposed slip

In this study, we investigate the problem of slip occurring at the interface of two fully dynamic poroelastic half-space, generally referred to as poroelastodynamic. Figure 1 shows the general setup of the problem.

Here we describe the off-fault response, for both static and dynamic fields, due to fault slip in the poroelastodynamic medium. The slip direction is in-plane, but otherwise, the slip is effectively arbitrary in both space and time; for example, we are not only solving for dislocation or a crack-like source. We apply an expansion in a spectral basis, which imposes periodic boundary conditions on the fault at the limits of the domain in *x* (i.e. the direction of slip on the fault). However, we solve the problem analytically for an infinite domain in *y* (i.e. normal to the slipping fault). We highlight that the poroelastic bulk is isotropic in terms of material properties, and the governing equations are linear. Thus implicitly, we assume infinitesimal strains everywhere except the interface.

## Theory

### Governing equations

The theory of quasi-static Biot poroelasticity (Biot 1941) in time and three-dimensional space can be compactly written as a set of four coupled partial differential equations and in terms of four field variables $u_i$ and *p*, where $u_i$ represents displacements in the *i*-th direction and *p* is the pore-pressure perturbation around an equilibrium (see Cheng 2016; Detournay and Cheng 1995, for general theory of the topic). The theory of poroelastodynamics (Biot 1956a, b, 1962) can be presented in a comparable manner, however, this representation results in six partial differential equation in terms of six field variables $u_i$ and $w_i$, where the latter represents the specific relative fluid to solid displacement (Cheng 2016). This adds considerable complexity to any numerical or analytical investigation compared to the quasi-static theory. The complexity is further amplified by the fact that imposing intuitive boundary conditions on the $w_i$ fields is challenging.

However, governing equations of poroelastodynamics are considerably simplified in the frequency domain (Cheng et al. 1991) where they can be presented in the more intuitive form of four equations and in terms of four field variables $u_i$ and *p*, similar to the quasi-static Biot poroelasticity. Further, such representation is also attained in the more general Laplace domain (Chen 1994), which is more appropriate for investigating initial value problems. Chen (1994) represented the governing equations as follows:

1 $$\begin{aligned}&({\lambda }+\mu ) \tilde{u}_{j, i j}+\mu \tilde{u}_{i, j j}-\alpha _{1} \tilde{p}_{, i}\nonumber \\&\quad -\rho _{1} s^{2} \tilde{u}_{i}+\tilde{f}_{i}=0, \end{aligned}$$

2 $$\begin{aligned}&\zeta \tilde{p}_{, i i}-\frac{s}{Q} \tilde{p}-\alpha _{1} s \tilde{u}_{i, i}+\tilde{\gamma }=0, \end{aligned}$$

where repeated indices represent a sum over the spatial dimensions. In 3D, $i = 1, 2,$ or 3, but for plane strain $i= 1$ or 2. Subscripted commas (e.g. $\tilde{p}_{, i}$) represent a derivative with respect to the *i*-th spatial dimension. There is an implicit assumption in Eqs. 1 and 2 that all fields are at equilibrium, or in other words zero, at time $t=0$.

The material parameters $\lambda$ and $\mu$ are the drained Lamé constant, with $\mu$ being the shear modulus, which is invariant of drained and undrained conditions. $f_i$ and $\gamma$ represent body forces and the rate of fluid injection respectively, but both are set to zero in this study. Here $\alpha _1 = \alpha - \rho _f s \zeta$, where $\alpha = 1 - K_D/K_S$ is the Biot’s coefficient with $K_D$ and $K_S$ representing the drained bulk modulus and the solid constituent bulk modulus. $\rho _f$ is the fluid density and $\zeta =((1 / \kappa )+m s)^{-1}$, where $\kappa$ is the fluid mobility (permeability over dynamic viscosity), $m=\rho _{f} / n$ (Zienkiewicz et al. 1980) with *n* representing porosity. Further, $\rho _{1}=\rho -\rho _{f}^{2} s \zeta$, where $\rho = (1-n)\rho _s + n \rho _f$ is the density of the combined fluid-solid phases with $\rho _s$ as being the density of the solid constituent. Finally $(1 / Q)=\left( n / K_{f}\right) +\left( (\alpha -n) / K_{S}\right)$ where $K_f$ is the bulk modulus of the fluid constituent.

The ~ sign represents a Laplace transformed variable, for example, in the case of the pore-pressure

3 $$\begin{aligned} \tilde{p}(s,x_i) = \int _0^{\infty } p(t,x_i) e^{-st} dt, \end{aligned}$$

where *s* is Laplace frequency parameter that generally has both non-zero imaginary and real parts.

Furthermore, we note Hooke’s law

4 $$\begin{aligned} \sigma _{i j}=\lambda u_{k, k} \delta _{i j}+\mu \left( u_{i, j}+u_{j, i}\right) -\alpha p \delta _{i j} , \end{aligned}$$

which has the same form as in quasi-static poroelasticity and provides a way to represent solutions of the governing equations in terms of stresses. We note that Hooke’s law has no explicit time-derivatives and is linear, so the Laplace transform is obtained trivially by adding ~ to the field variables. Table 1 lists the parameter values used in this study, which are kept constant unless otherwise stated. The choice of parameters represents a generic rock and water phase; however, we stress that considerable variability for most poroelastic parameters is observed for different types of rocks (Cheng 2016). Other parameters, not listed in the table, can be computed based on the values in the table.

**Table 1:** List of parameters kept constant unless otherwise specified

Parameter	Definition	Value
$\lambda$	Lamé’s first parameter (drained)	30.0 GPa
$\mu$	Lamé’s second parameter (Shear modulus)	30.0 GPa
$\alpha$	Biot coefficient	0.5
*n*	Porosity	0.05
$\rho _f$	Fluid density	1000 kg/m$^3$
$\rho$	Fluid and solid phases mixture density	3000 kg/m$^3$
$\kappa$	Mobility (permeability over dynamic viscosity)	3.333 $\cdot$ 10$^{-14}$ m$^2$/(Pa s)
$K_f$	Bulk modulus of the fluid	2.1 GPa

### Spectral boundary integral solutions

Here we describe the procedure to obtain the spectral boundary integral solutions. This section shows that all off-fault fields can be represented as a convolution of the slip speed and a kernel function.

First we shall reduce to governing Eqs. (1 and 2) to the plane strain case. This is done trivially by only having the indexes span $i = 1,2$. For more transparency, in the equations to follow we shall refer to the $i=1$ index as the *x* dimension and $i=2$ as the *y* dimension as in Fig. 1.

The first step is Fourier transforming in *x*, thus now we have applied a joint Fourier-Laplace transform, for example to the pore-pressure:

5 $$\begin{aligned} \hat{\tilde{p}}(s, k , y)=\int _{0}^{\infty } \int _{-\infty }^{\infty } p(t, x, y) e^{-i k x-s t} d x d t. \end{aligned}$$

In this dual transform domain, one can show that the governing Eqs. 1 and 2 reduces to

6 $$\begin{aligned}&\mu \hat{\tilde{u}}_{x,yy} = \left( (\lambda + 2\mu ) k^2 + \rho _1 s^2 \right) \hat{\tilde{u}}_{x} - (\lambda + \mu ) i k \mu \hat{\tilde{u}}_{y,y} + \alpha _1 \mu \hat{\tilde{p}} \end{aligned}$$

7 $$\begin{aligned}&(\lambda + 2 \mu ) \hat{\tilde{u}}_{y,y y} = -(\lambda + \mu ) i k \hat{\tilde{u}}_{x,y} + (\mu k^2 + \rho _1 s^2) \hat{\tilde{u}}_{y} + \alpha _1 \hat{\tilde{p}}_{,y} \end{aligned}$$

8 $$\begin{aligned}&\zeta \hat{\tilde{p}}_{,y y} = \alpha _1 s i k \hat{\tilde{u}}_{x} + \alpha _1 \hat{\tilde{u}}_{y,y} + (\zeta k^2 + s/Q) \hat{\tilde{p}}_{,y} \end{aligned}$$

At this stage, the solution strategy is straightforward but tedious. First, the second derivatives with respect to *y* must be eliminated using the standard method of treating the first-order derivative as a separate function, thus introducing three more equations into the problem. The system of governing equations can thus be represented as

9 $$\begin{aligned} \frac{d}{d y} \mathbf{f} = \mathbf{A} \mathbf{f} \end{aligned}$$

where $\mathbf{f} = [\hat{\tilde{u}}_x,\hat{\tilde{u}}_{x,y},\hat{\tilde{u}}_y,\hat{\tilde{u}}_{y,y},\hat{\tilde{p}},\hat{\tilde{p}}_{,y}]^T$ is the vector of relevant field variables and their derivatives, which are a byproduct of reducing the system of equations to the first order. $\mathbf{A}$ is a 6x6 matrix and its elements can be determined from Eqs. 6, 7, and 8.

In other words, we have obtained an equivalent system of six first-order linear ordinary differential equations, which can be solved in a standard manner by computing eigenvalues and eigenvectors of $\mathbf{A}$. We do not show this step in this paper since it is carried out with Matlab’s symbolic manipulator toolbox (The MathWorks 2019).

Each one of the six eigenvectors introduces an unknown coefficient which must be determined by imposing boundary conditions. We impose boundary conditions at $y = 0$ and need a separate solution for the upper half-space and the lower half-space, thus resulting in a total of 12 unknowns. The boundary conditions are as follows.

10 $$\begin{aligned} \begin{aligned}&\lim _{y \rightarrow \pm \infty } \hat{\tilde{u}}_{x}^{\pm }=0, \\&\lim _{y \rightarrow \pm \infty } \hat{\tilde{u}}_{y}^{\pm }=0, \\&\lim _{y \rightarrow \pm \infty } \hat{\tilde{p}}^{\pm }=0, \\&\lim _{y \rightarrow 0^{\pm }} \hat{\tilde{u}}_{x}^{+}-\hat{\tilde{u}}_{x}^{-}= \hat{\tilde{\delta }} \\&\lim _{y \rightarrow 0^{\pm }} \hat{\tilde{u}}_{y}^{+}-\hat{\tilde{u}}_{y}^{-}=0 \\&\lim _{y \rightarrow 0^{\pm }} \hat{\tilde{p}}^{\pm } = 0, \\&\lim _{y \rightarrow 0^{\pm }} \hat{\tilde{\sigma }}_{x y}^{+}-\hat{\tilde{\sigma }}_{x y}^{-}=0, \\&\lim _{y \rightarrow 0^{\pm }} \hat{\tilde{\sigma }}_{y y}^{+}-\hat{\tilde{\sigma }}_{y y}^{-}=0, \end{aligned} \end{aligned}$$

where we indicated a field in the upper half-space $(y>0)$ with a superscript $^+$ and the lower half-space $(y<0)$ with superscript $^-$ (See Fig. 1 for reference). The first three statements listed (corresponding to six equations) guarantee that all fields decay at infinity. These conditions are first applied by setting coefficients that scale terms that diverge at $y \rightarrow \pm \infty$ to zero, thus assuming that all fields go to zero at infinite distance away from the fault and reducing the resulting unknowns to six. At this stage the solution, without having imposed the last 6 boundary conditions, can be written as

11 $$\begin{aligned} \mathbf{g} = \mathbf{V} \mathbf{d} \end{aligned}$$

where $\mathbf{d} = \mathbf{c} \cdot \mathbf{e} = [c_1 e^{E_1 y},c_2 e^{E_2 y},c_3 e^{E_3 y},c_4 e^{E_4 y},c_5 e^{E_5 y},c_6 e^{E_6 y}]^T$, $c_n$ being the $n-$th coefficient that needs to be determined by the interface condition in Eq. 10 and $E_n$ is the *n*-th eigenvalue of $\mathbf{A}$ for upper and lower half-spaces once removing the eigenvalues that cause fields to diverge at infinity (by setting the corresponding coefficient to zero). The relevant fields are expressed in vector $\mathbf{g} = [\hat{\tilde{u}}_x^+,\hat{\tilde{u}}_{x}^-,\hat{\tilde{u}}_y^+,\hat{\tilde{u}}_{y}^-,\hat{\tilde{p}}^+,\hat{\tilde{p}}^-]^T$. The matrix $\mathbf{V}$ contains the eigenvectors corresponding to each eigenvalue in each column, which are computed from $\mathbf{A}$.

The latter 5 boundary condition statements (six equations) are interface conditions of the half-space boarders at $y = 0$. First, we assume an arbitrary displacement discontinuity $\delta$, also known as slip, can occur at the interface. Second, we state that the interface cannot open or close in on itself. Third, that the pore pressure at the interface is zero, we highlight that in many cases, this may not be an appropriate boundary condition for slip problems in poroelastic solids if the symmetry of the problem is broken or the fault interface has a finite width (see Heimisson et al. 2021, for discussion). In our mathematical problem, the compressional lobe on one side of the fault is equal but of opposite sign to the dilational lobe on the other and thus the pore pressure is of equal and opposite sign, furthermore, the fault width is infinitesimal such that discontinuity in pore pressure can be instantaneously resolved by diffusion. Thus by virtue of this anti-symmetry, the pore pressure change will be zero. Natural faults are much more complex and we do no expect such strict conditions to hold. However, in this study, we are simulating the off-fault fields at some observation plane $y = y_o$ due to imposed slip history, and thus we do not expect this condition to be as important as, for example when understanding the frictional stability of the fault. The last two boundary condition statements impose continuity of traction across the interface.

The implementation of the boundary conditions can be presented as a linear system of equations.

12 $$\begin{aligned} \mathbf{b} = \mathbf{G} \mathbf{c}, \end{aligned}$$

where $\mathbf{b} = [\hat{\tilde{\delta }},0,0,0,0,0]^T$. Thus $\mathbf{c} = \hat{\tilde{\delta }} \mathbf{G}_{:,1}^{-1}$, where $\mathbf{G}_{:,1}^{-1}$ being the first column of the inverse of $\mathbf{G}$.

Now the solutions vector $\mathbf{g}$ can be fully determined.

13 $$\begin{aligned} \mathbf{g} = \hat{\tilde{\delta }} \mathbf{V} \left( \mathbf{G}_{:,1}^{-1} \cdot \mathbf{e} \right) , \end{aligned}$$

stresses and strains can be obtained from 13 using Hooke’s law (Eq. 4) and the appropriate derivatives. Equation 13 shows that in the Laplace domain all fields are multiplied by the fault slip $\hat{\tilde{\delta }}$. Using the convolution theorem of Laplace transforms we can invert the transform by turning it into a convolution in the time domain.

14 $$\begin{aligned} \mathcal {L}^{-1} \left( \mathbf{g} \right) (t) = \int _0^t \hat{\delta }(t') \mathcal {L}^{-1} \left( \mathbf{V} (\mathbf{G}_{:,1}^{-1} \cdot \mathbf{e} ) \right) (t-t') dt'. \end{aligned}$$

If $|y_o| > 0$ (see Fig. 1) there is no instantaneous response between slip and observed fields at $y = y_o$, otherwise causality would be violated, so integration by parts renders a different expression:

15 $$\begin{aligned} \mathcal {L}^{-1} \left( \mathbf{g} \right) (t) = \int _0^t \hat{v}(t') \mathcal {L}^{-1} \left( \frac{1}{s} \mathbf{V} (\mathbf{G}_{:,1}^{-1} \cdot \mathbf{e} ) \right) (t-t') dt', \end{aligned}$$

where the solution is provided as a convolution in terms of the slip speed ($\dot{\delta } = v$). We prefer this representation for reasons discussed in Sect. 2.4.

We may write more explicitly, for example, the pore-pressure in the upper half-space as

16 $$\begin{aligned} \hat{p}^+(y,t) (k,y,t) = \int _0^t \hat{v}(k,t') K^{p+} (k,y,t-t') dt', \end{aligned}$$

where $K^{p+}$ is inverse Laplace transform of the 5th element in the column vector $\left( \frac{1}{s} \mathbf{V} (\mathbf{G}_{:,1}^{-1} \cdot \mathbf{e} ) \right)$. Another example:

17 $$\begin{aligned} \hat{u}_x^+(y,t) (k,y,t) = \int _0^t \hat{v}(k,t') K^{u_x +} (k,y,t-t') dt', \end{aligned}$$

where $K^{u_x +}$ is inverse Laplace transform of the first element in the column vector $\left( \frac{1}{s} \mathbf{V} (\mathbf{G}_{:,1}^{-1} \cdot \mathbf{e} ) \right)$.

In summary, we have shown that all fields can be represented as a convolution of the slip speed and a convolution kernel that needs to be determined.

We end this section by making a few remarks about the convolution kernels.

1. Depending on if the field in question is symmetric or anti-symmetric, the upper and lower half-space kernels are either the same or differ in sign.
2. The kernels need to be determined by numerically inverting the Laplace transform since analytical inversion has not been feasible due to the extreme complexity of the expressions, see Sect. 2.4 for discussion.
3. Each kernel is a function of time, the distance from the fault $y = y_o$ (since $\mathbf{e} = [e^{E_1 y},e^{E_2 y},e^{E_3 y},e^{E_4 y},e^{E_5 y},e^{E_6 y}]^T$), the wavenumber *k*, and the governing material parameters introduced in Eqs. 1 and 2.
4. Each convolution kernel is independent on the slip history, thus once computed it can be applied to any slip history provided that spatial and temporal discretization resolves the rupture process.

### Inversion of fourier transform

The inversion of the Fourier transform is carried out by expanding the slip speed in a Fourier basis or, in other words, a Fourier series:

18 $$\begin{aligned} v (x,t) = \sum _{n = -N/2}^{N/2 - 1} V_{n}(t) e^{ik_n x}, \quad k_n = \frac{2 \pi n}{L}, \end{aligned}$$

where *L* is the domain size, and *N* is the number of discrete and evenly spaced points in the domain. $V_n(t)$ is the *n*-th Fourier coefficient corresponding to a discrete wavenumber of $k_n$. Computation of the Fourier coefficients is done efficiently using the fast Fourier transform algorithm (FFT). Thus from Eq. 17 we can obtain a mapping between the n-th Fourier coefficient of *v*(*x*, *t*) defined at $y=0$ and the n-th Fourier coefficient of $u_x^+(x,t)$ evaluated at observation plane $y=y_o$ (Fig. 1)

19 $$\begin{aligned} U_x^{n+} ({k_n,y=y_o,t}) = \int _{0}^t V_n(k_n, t') K^{u_x+} (y=y_o, t -t',k_n) dt', \end{aligned}$$

then the corresponding displacements can be computed for the entire observation plane:

20 $$\begin{aligned} u_x^+ (x,y=y_o,t) = \sum _{n = -N/2}^{N/2 - 1} U_{i}^{n+}(t) e^{-ik_n x}, \quad k_n = \frac{2 \pi n}{L}, \end{aligned}$$

but this step can be done efficiently with the inverse fast Fourier transform algoritm (iFFT).

Similarly, we may compute the pore-pressure at observation plane $y = y_o$ by using the following mapping between the Fourier coefficients of the slip speed and the Fourier coefficients of the pore-pressure:

21 $$\begin{aligned} P^{n+} ({k_n,y=y_o,t}) = \int _{0}^t V_n(t') K^{p+} (y=y_o, t -t',k_n) dt', \end{aligned}$$

and the pore-pressure is computed

22 $$\begin{aligned} p (x,y=y_o,t) = \sum _{n = -N/2}^{N/2 - 1} P^{n+} (t) e^{-ik_n x}, \quad k_n = \frac{2 \pi n}{L}. \end{aligned}$$

Any other relevant field, either stress or strain, can be then treated in the same way by applying the appropriate derivatives of the relevant kernels and superimpose them. We highlight that spatial derivatives of the kernels with respect to *x* are carried out trivially by multiplying the kernel by *ik*.

### Numerical approach

While the bulk of the method presented is based on analytical analysis, the final steps in obtaining the convolution kernels and then simulating various field are carried out numerically. The procedure is as follows:

1. Given a set of material parameters, such as $\lambda$, $\mu$, $\alpha$, etc., we compute $\left( \frac{1}{s} \mathbf{V} (\mathbf{G}_{:,1}^{-1} \cdot \mathbf{e} ) \right)$ using Matlab’s symbolic manipulator.
2. We define a fault length *L* and spatial discretization $\Delta x$, here taken as 200 m and 0.5 m respectively and compute the corresponding array of wavenumbers $k_n$. Further, we define the simulation time and time-steps, here 0.03 s and 5$\cdot$10$^{-5}$ s respectively, where the time-steps are evenly spaced. Time-step discretization means that a P-wave will take two time-steps to approximately propagate the distance of $\Delta x$, note however that P-waves are dispersive and do not have a single wave speed (e.g. Cheng 2016).
3. We set $y=y_o$, and in this study, we explore values of 5 m, 10 m, 20 m.
4. We numerically evaluate the inverse Laplace transform at each wavenumber and time-step pair, for example, in Eq. 20. Thus the convolution kernel can be represented as a discrete 2D matrix where each column is a time-step, and each row corresponds to a wavenumber.
5. Given a prescribed slip speed history *v*(*x*, *t*), FFT is used to compute the Fourier coefficients, then the convolution in time is carried out using the trapezoidal rule, and iFFT is used to construct the desired field at $y = y_o$

The 4-th step above is by far the most numerically expensive and non-trivial, and thus it is worth discussing more. To invert the Laplace transform, we use the well-known Talbot contour integration (Talbot 1979) to improve the convergence of the Bromwich integral. We use the contour parameters identified by Abate and Valko (2004) and follow their algorithm exactly where the contour is discretized into $N_{LP}$ intervals, and then the integral is computed with the trapezoidal rule as is standard due to its exponential convergence in contour integration (Trefethen and Weideman 2014). In this case, we expect convergence of the integral should be no worse than $\sim 1/\sqrt{N_{LP}}$, but the convergence may depend on the function to be inverted, how well suited the selected contour function is for that function, and the selection of contour parameters for each particular case (e.g. Weideman 2006; Dingfelder and Weideman 2015).

The challenging aspect of inverting the Laplace transform is that one may need to evaluate the contour integral at a much higher precision than typical double precision. Indeed for the Talbot method, the number of significant digits needed to compute the contour integral is approximately $0.6 N_{LP}$ thus, one can expect an inaccurate inversion of the Laplace transform using double precision if higher order than $N_{LP} = 25$ is needed. If a function contains high frequencies, for example, for high-frequency waveforms, this function will need a longer contour to be inverted. Intuitively, this occurs because this function contains non-zero values far from the real axis, which represent the high-frequency content. When exploring the convergence of the inverse Laplace transform in this study with respect to $N_{LP}$ we observed that the slip-speed convolution kernels $\frac{1}{s} \mathbf{V} (\mathbf{G}_{:,1}^{-1} \cdot \mathbf{e} )$ compared to the slip convolution kernels $\mathbf{V} (\mathbf{G}_{:,1}^{-1} \cdot \mathbf{e} )$ had improved convergence. This is because the scaling of 1/*s* causes faster decay in the complex plane. Nevertheless, we concluded that we required $N_{LP} = 200$ to obtain acceptably well-resolved results for the problems setup, spatial discretization and material parameters. We thus needed to compute the contour integral with 130 significant digits, but we used $0.65 N_{LP}$ to be on the safe side. This is possible with Matlab by treating the discretized contour integral as a symbolic expression and then finally evaluate the expression at the desired precision, which can be done with Matlab’s vpa function. While this allows for computing the inverse Laplace transform at virtually any desired precision, this is a very computationally expensive. Computing one element in the pore-pressure kernel at $N_{LP} = 200$ takes about 30 s, but based on numerical exploration, it appears that the computation time scale approximately linearly with $N_{LP}$. In this study, the kernels have $400 \cdot 600 = 240000$ elements, but only half the elements are needed after utilizing symmetries, or anti-symmetries, with respect to the wavenumber. Thus computing a single kernel on a single core takes about 40 days. However, all elements of the kernel matrix are independent, and thus, the computational time is primarily only limited by how many cores can be used for the computation. In this study, we used 144 cores to compute each kernel and gained 144 fold speedup in the computation by using a straightforward parfor loop parallelization in Matlab.

Once a kernel has been computed, then carrying out the convolution in step 5 can be done on a single core with a non-optimized code in a few seconds. We thus highlight that the vast majority of the time required goes into computing the kernel, but once that is done. A large number of simulations with arbitrary slip speed histories (as long as they agree with the discretization) can be carried out rapidly. The method, therefore, offers an opportunity to explore different slip speed distributions at perhaps unprecedented speed for numerical methods that can simulate static and dynamic fields in a poroelastodynamic solid. However, the method is prefaced with a computationally intensive kernel building.

#### Source models used in this study

**Fig. 2:**
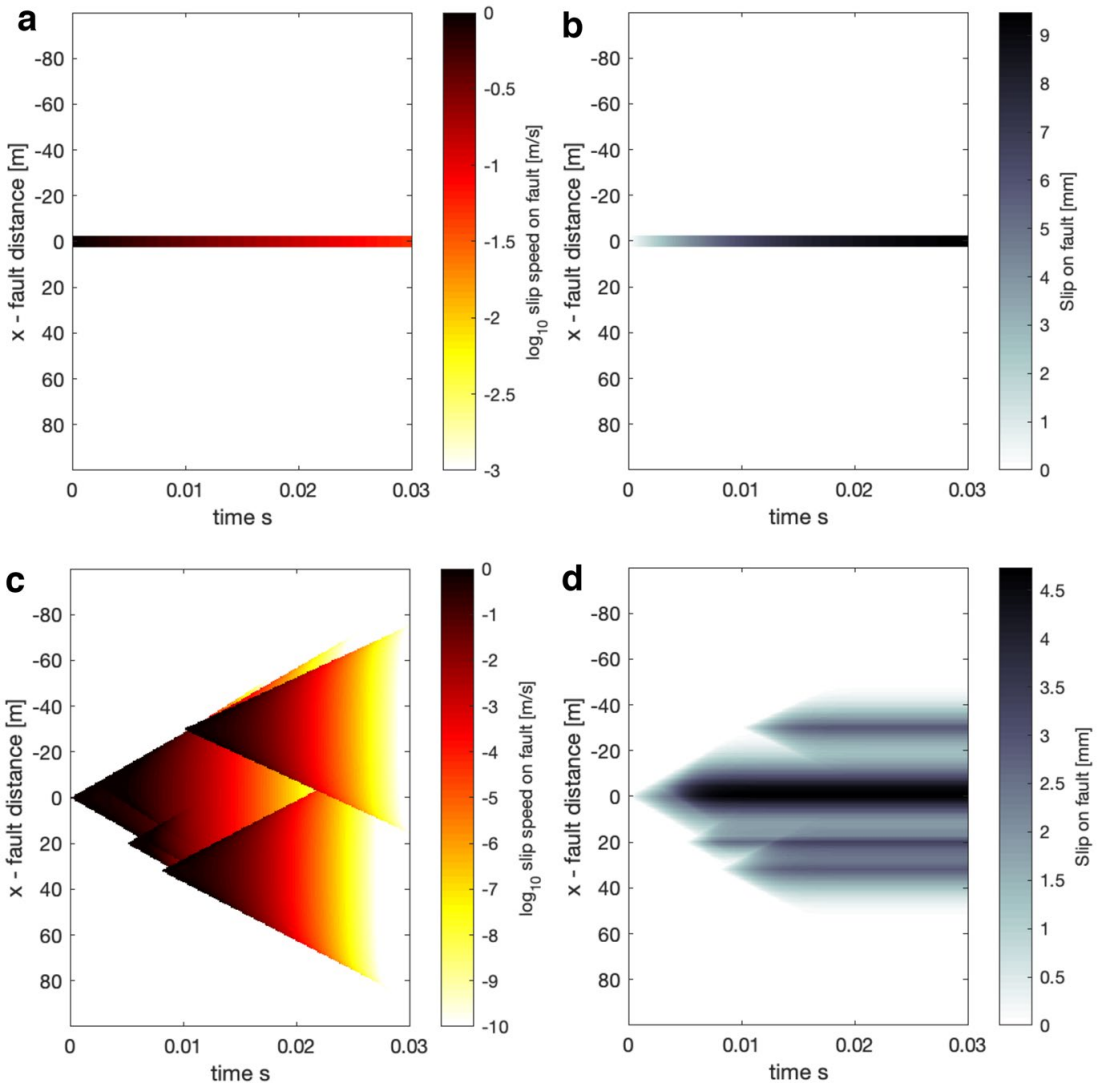
Illustration of the source properties (located at y=0) used in the study both in terms of slip speed and cumulative slip. The source duration is 0.03 s and is discretized into time-steps of 5$\cdot$10$^{-5}$ s (same as the convolution kernels) . **a** shows the simple dislocation source slip speed. **b** shows the simple dislocation source slip. **c** shows the complex multiple crack near-field source slip speed. **d** shows the complex multiple near-field crack source slip

As has been discussed, once the kernel has been computed, the source model (slip rate history in time and space) can be selected arbitrarily, and the relevant fields at $y=y_o$ can be constructed with minimal computational time and resources. The source model is assumed to have the same time discretization as the convolution kernels, that is a duration of 0.03 s and time-steps with length of 5$\cdot$10$^{-5}$ s. To narrow the focus in this study, we shall select two source models to highlight two different regimes: first, a simple dislocation source with an exponential time-dependence with a characteristic rise time of 0.01 and a total slip of 0.01 m. The total source dimension is 5 m, and we will both observe wave-mediated and quasi-static fields in the intermediate distance to far-field range. The second, a complex near-field source constructed by several self-similar propagating cracks (e.g. Burridge and Willis 1969) that are activated at different times and locations and the rupture speeds set to be around 90 % of the S-wave speed in an elastic solid with the same density and shear modulus. We introduce a small variability of the rupture speeds within 10% difference for the fastest and slowest. The self-similar crack ruptures are terminated by multiplying a time-dependent factor, which is a half-Gaussian with a standard deviation of either 0.005 or 0.00025 s. Since the self-similar crack has a singular slip rate at the propagating front, we regularize the model by capping the slip rate at 1 m/s, thus effectively introducing a cohesive zone. The complex source has a final dimension of about 80 m, and thus the response at $y = y_o$ can be interpreted as the near-field response. We highlight that the complex near-field source, as well as the simple dislocation source, are not necessarily realistic examples of earthquake ruptures at different scales and are simply selected to illustrate potential regimes. Figure 2 offers a visualization of the sources showing both slip speed and slip.

The spatial and temporal discretization of the kernels is important in terms of resolving the bulk response from a given source model. As we mentioned before, the minimum spatial grid size is set to 0.5 m and the time-step to $5 \cdot 10^{-5}$ s. First, the spatial discretization was determined approximately such that the minimum length scale of the source model was resolved. For the simple source model this is the 5 m length of the source. However, the complex source is a superposition of self similar cracks that nucleate from a point with singular slip speed at the rupture tip. In principle, such a rupture source has no cohesive zone and thus infinitesimally small minimum length scale. However, we capped the slip speed at 1 m/s and thus effectively introduced a cohesive zone, but since the source nucleates at a point this scale will not be resolved early on. The minimum time-step, as was mentioned earlier, was estimated given the spatial discretization based on resolving the propagation of the P-wave from one grid point to another. We carried out tests where we computed kernels with higher spatial and/or temporal resolution for $N_{LP} = 200$ and compared to the grid size of 0.5 m and the time-step of $5 \cdot 10^{-5}$ s. This error was determined to be negligible compared to the numerical error from inversion of the Laplace transform (see Sect. 3.2 for more discussion on convergence with $N_{LP}$).

## Results

In this results section, we apply the method presented in previous sections to investigate several problems related to earthquake physics and simulations of earthquakes and possible near-field or intermediate distance observations. Further, we explore some aspects of the numerical implementation.

First, we explore and visualize several fields for a reference case. Second, we present a Kernel convergence study to provide more insight into the robustness of the numerical inversion of the Laplace transform. Third, we investigate some of the expected characteristics if the pore pressure is observed at a high rate relatively close to an earthquake source. Finally, we ask the question, is accounting for poroelastodynamic effects needed when investigating earthquake signals and interaction, or can we approximate these effects with a simpler elastic theory with an undrained one-way coupling of strain and pore-pressure? We shall refer to the full poroelastodynamic simulation as “coupled” and the elastic simulation with one-way pore-pressure coupling as “decoupled” for short.

### Reference case results

**Fig. 3:**
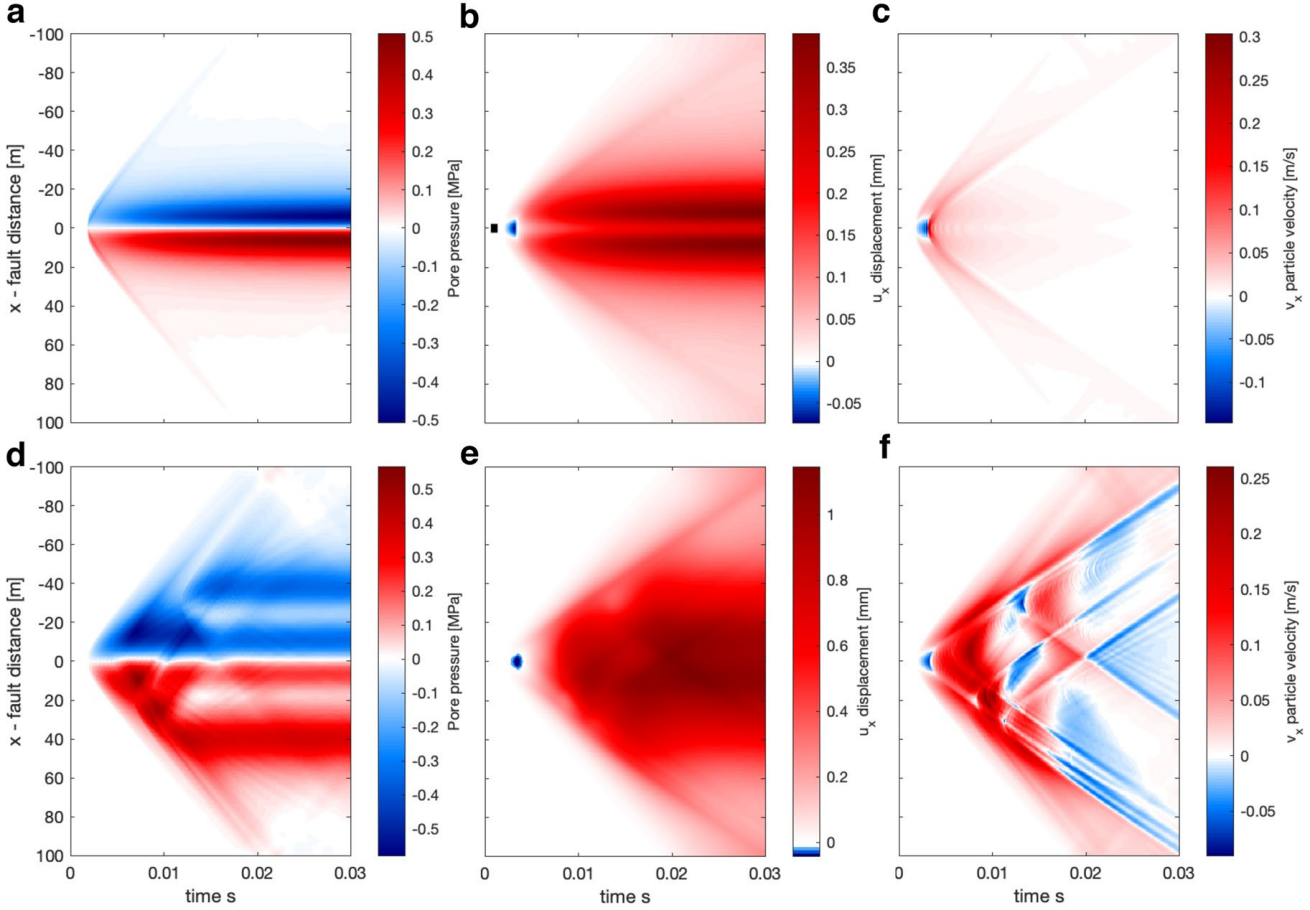
Reference case for the source models in Fig. 2 at $y_o = 10$m. Top row represents results for the simple source, the bottom row for the complex source. Panels **a, d** show pore-pressure change, panels **b, e** show displacements in slip parallel direction, and panels **c, f** show particle velocities in slip parallel direction

We start by presenting a reference case with $y_o = 10$m, for the simple and complex source (see Fig. 3).

Figure 3 shows well that within the time-frame of the simulations, we observe both the wave-mediated response as well as the realization of the static or quasi-static response. For example, panel **a** shows the P-wave induced pore-pressure response, as well as the growth of the two lopes in the $\pm 20$ m range, which represent the pore-pressure response predicted by the quasi-static theory and is mostly realized in the time range of 0.02-0.03 s. Panel **b** clearly shows the S wave arrival and propagation, which induces no pore-pressure response and is thus not seen in panel **b**. As is expected, it is more difficult to identify features in the complex near-field source. However, a comparison of the top and the bottom row shows some general similarities, for example, displacements in the opposite direction of slip before the arrival of the S wave (**b, e**).

### Kernel convergence study

**Fig. 4:**
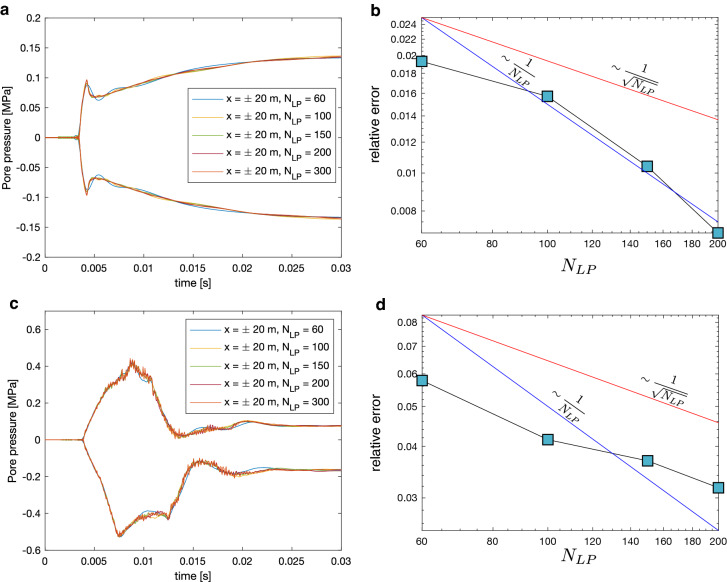
Convergence test of the dynamic pore-pressure with increasing $N_{LP}$. **a** pore-pressure profiles with time at $\pm 20 m$, with varying $N_{LP}$ for the simple dislocation source shown in Fig. 2. Visually speaking, the agreement is good, although some difference is observed in $N_{LP} = 60$. We note that for $N_{LP}$ values less than 60 the agreement deteriorates rapidly. Panel **b** shows change in relative error with increasing $N_{LP}$, the relative error is defined as the $L_1$ norm of the residuals of the $N_{LP}$ solution (indicated by the horizontal axis) and the $N_{LP} = 300$ solution divided by the $L_1$ norm of the latter **a**, or mathematically $||p^+(x = \pm 20, t, N_{LP}) - p^+(x = \pm 20, t, 300) ||_1 / || p^+(x = \pm 20, t, 300) ||_1$. We observe approximately $1/N_{LP}$ convergence. Panel **c** corresponding plot to **a** but for the complex source, here we observe higher frequencies associated with the propagation of the crack tip. **d ** shows convergence of the complex source. We observe a slower convergence that is more similar to $1/\sqrt{N_{LP}}$, we suggest that this is due to high frequency content

Due to the many nuances of computing numerically the inverse Laplace transform, we shall here report a convergence test with respect to $N_{LP}$ (Fig. 4). In this test, we explore the convergence of the pore-pressure, we explore the convergence of the pore-pressure because our exploration seems to suggests that it requires higher $N_{LP}$ to reach an acceptable error compared to other fields. The reason for this is likely that the pore pressure depends on the volumetric stress, which in turn depends on the derivatives of displacements fields. Due to this dependence of various derivatives, the pore pressure may contain shorter wavelengths and thus higher frequencies.

A visual inspection of Fig. 4 suggests that at a contour discretization with $N_{LP} = 100$ renders acceptable results. However, in the case of the complex source, we observe significant relative error due to the excitation of higher frequencies. We have thus chosen $N_{LP} = 200$ in the study and in the results. We stress that the simple and complex sources in Fig. 4 are produced with the same convolution kernels for each $N_{LP}$ value. It may thus be surprising that the two results have different accuracy and convergence. However, we observe that a low order kernel (with low $N_{LP}$) can give an accurate result if it is convolved with a function that doesn’t contain high frequencies since the higher frequencies are not correctly represented in the kernel will be averaged out. We postulate that there should be a relationship between $N_{LP}$ and the maximum frequency one wishes to simulate, but we leave this to future work.

### Pore-pressure evolution with distance

Here we explore in more detail the characteristics of the dynamic and static pore-pressure fields. We refer to the dynamic pore-pressure as the wave-mediated changed, which are not predicted by a non-inertial theory. The static response is the poroelastic response at short distances ($|x| < 20$ m), which correspond to the undrained change of the quasi-static poroelastic theory once wave mediated transfer of stresses has occurred (approximately at 0.02 s in most examples).

**Fig. 5:**
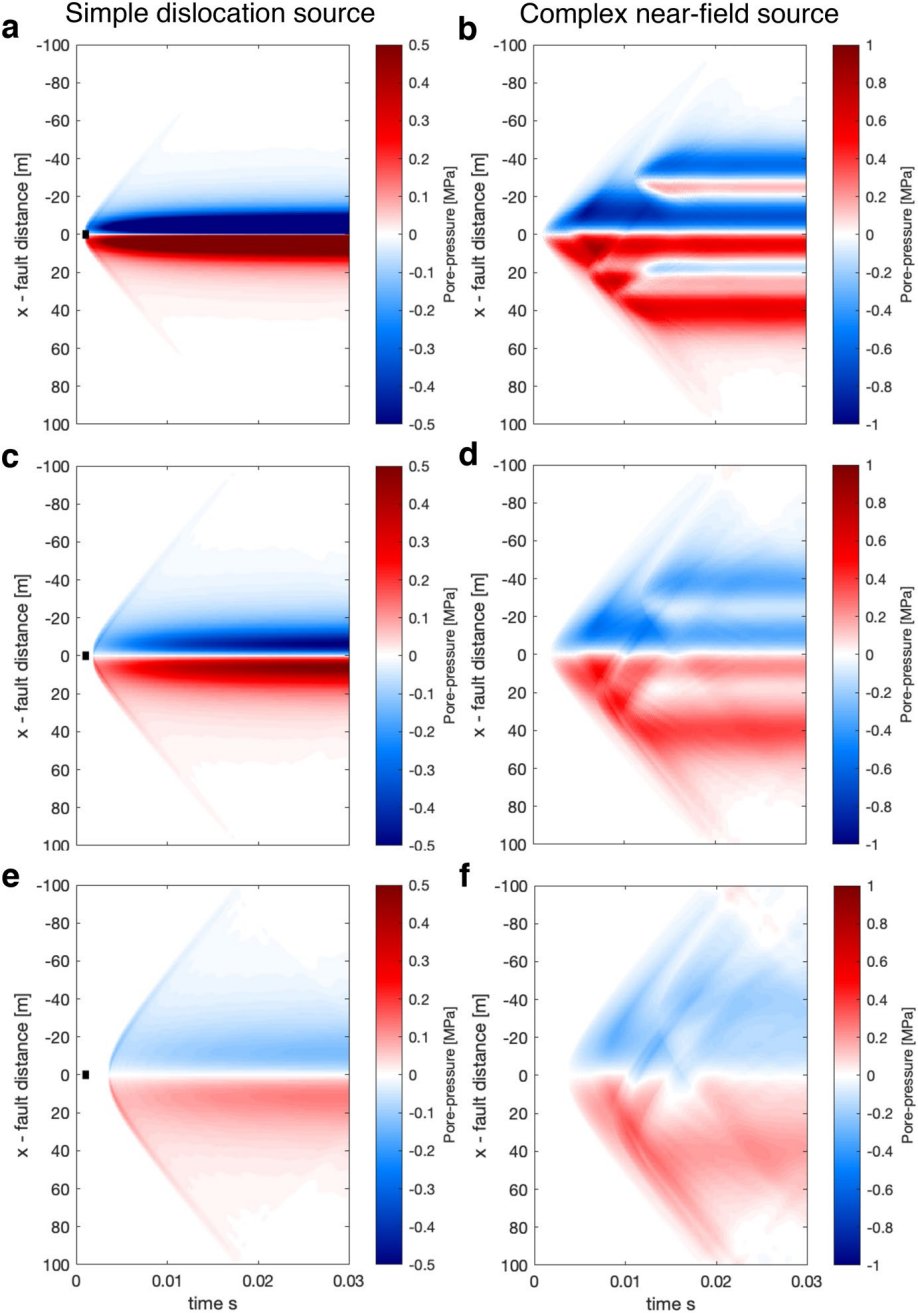
Visualization of the dynamic pore-pressure fields for both the simple source (left) and the complex source (right) at observation planes at varying distances: for **a, b** $y_o = 5$ m, for **c, d** $y_o = 10$ m, and for **e, f** $y_o = 20$ m

Perhaps, the most interesting result from simulating the dynamic pore-pressure is that the P-wave carries pore-pressure change over a distance much larger than the source dimension. For example, in Fig. 5e at the distance of $y_o = 20$ m, which is 4 times the source dimension. The static poroelastic response at short distances ($|x| < 20$ m) and the dynamic response are of similar magnitude. However, the dynamic response is carried much further parallel to the fault and maintains a significant value all the way to the boundary. We notice, also in the line-plots in Fig. 4, that the arrival of the P-wave is associated with a peak in pressure. Whether this peak is positive or negative depends on if the observation point is in the compressional or dilational area of the P-wave compared to the seismic source. The pressure decreases in magnitude once the P-wave has passed and either stabilize at a lower magnitude (in an absolute sense) or increases again if close enough to be affected by the quasi-static response.

In the complex source pore pressure, we observe some distinct characteristics. First, there are areas where positive pore-pressure change occurs in a predominantly negative pore-pressure area and vice-versa (Fig. 5b). However, as you move further away **d,f** the sign changes, this suggests that in the near-field of a seismic source, the pore-pressure can be complex and possibly difficult to interpret.

### Comparison of fully coupled and decoupled simulations

We now investigate if we can approximate the poroelastic effects, which results from the two way coupling of strain and pore pressure, with a decoupled representation. In the governing equations (Eqs. 1 and 2) we observe decoupling of Eqs. 1 and 2 if $\alpha = 0$ and $\rho _f = 0$. In this case Eq. 1 simply become the elastic wave equation with density $(1-n)\rho _s$. Similarly Eq. 2 simply becomes a diffusion equation. Since we don’t impose any changes in the pore pressure in the decoupled case, it will not change. In contrast, quasi-static poroelasticity only requires setting $\alpha = 0$ to decouple the elastic deformation and pore-pressure. Further, analysis of quasi-static poroelasticity provides a relationship between undrained pore pressure change and the volumetric stress (Rice and Cleary 1976)

23 $$\begin{aligned} p^{un} = -B \frac{\sigma _{kk}}{3}, \end{aligned}$$

where *B* is Skempton’s coefficient, which here is 0.37 given the parameters in Table 1.

**Fig. 6:**
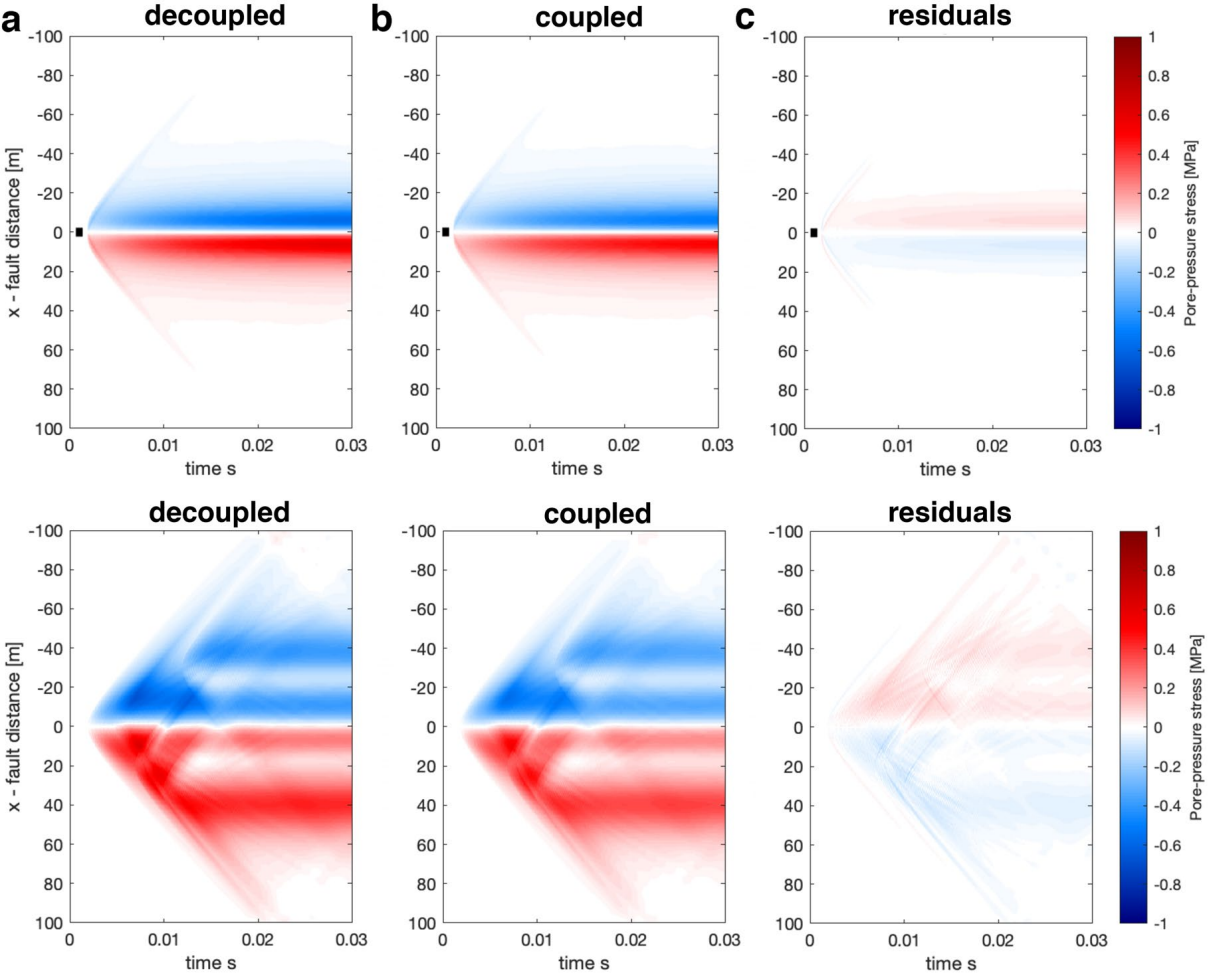
Comparison of pore-pressure response for the a decoupled simulation (**a**), coupled simulation (**b**), and residuals of coupled–decoupled (**c**). In the decoupled simulations (**a**) the pore pressure is computed using Eq. 23 after the simulations has been carried out. Top row shows the response for the simple source and the bottom row the complex source

First we explore if we may reasonably well approximate the pore-pressure response using Eq. 23 by comparing the pore pressure response at $y_o = 10$m for both a complex near-field seismic source and for simple dislocation source (see Fig. 2). The comparison is presented in Fig. 6.

Our results suggest that one can quite accurately approximate the dynamic pore pressure response using the decoupled method where only the elastic wave equation is solved, and then the pore-pressure response is computed with Eq. 23 after the simulation has been carried out. Here we have focused our attention on the short time scale, but we stress that at longer time scales, the decoupled and coupled approaches diverge as diffusion of the pore pressure becomes relevant.

To understand event clustering and fault interactions in induced seismicity settings, as well as other cases, we investigate the dynamic stresses on faults of different orientations, specifically the Coulomb stress (with the coefficient of friction set to 0.6) and the effective normal stress. Since the Coulomb and effective normal stresses incorporate several components of the strain and the pore-pressure, we suggest that if there are significant differences observed in any of the relevant fields, that should be revealed by investigating the Coulomb and effective normal stresses.

**Fig. 7:**
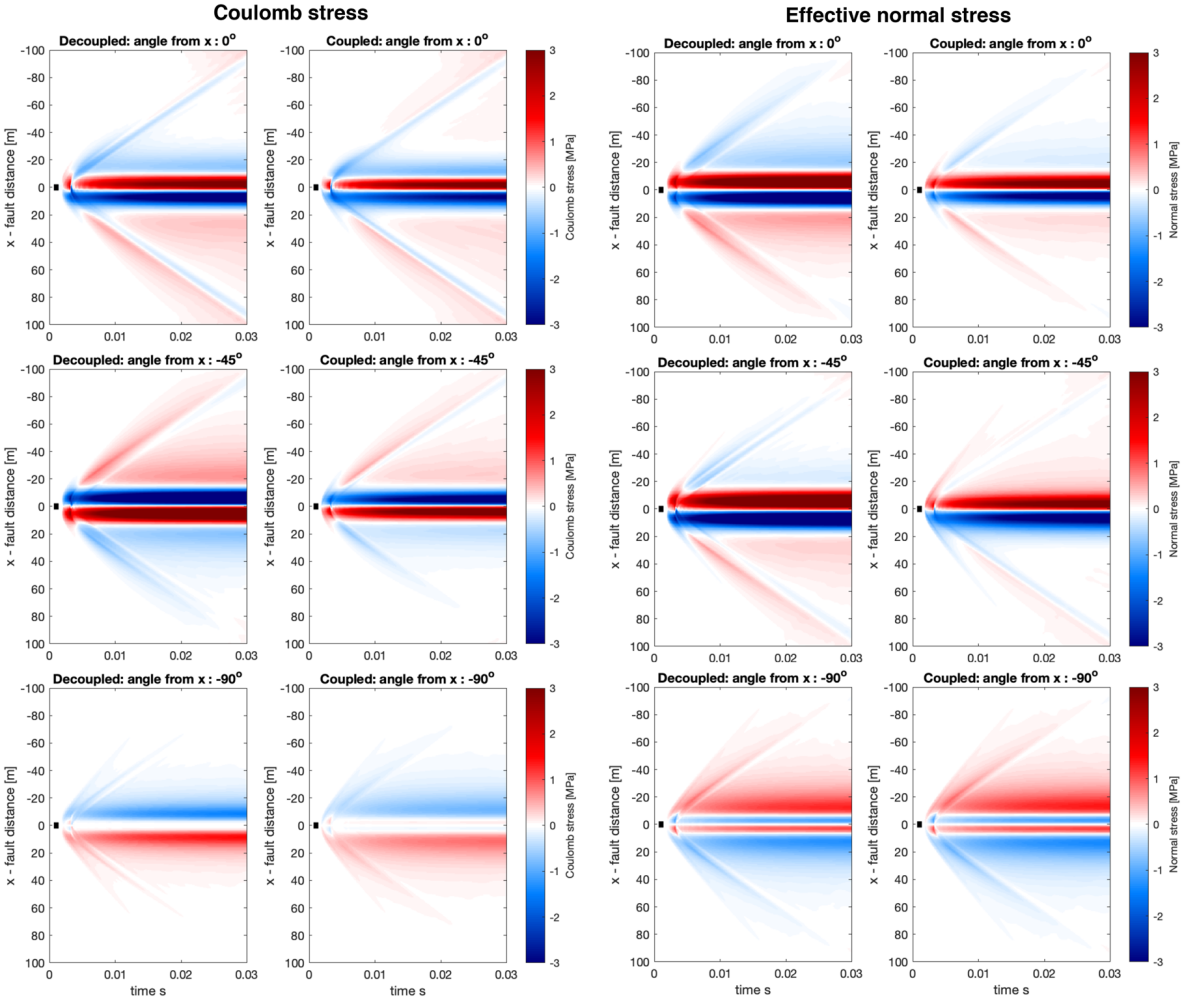
Coulomb stresses (left) and effective normal stress (right) calculated on $y_o = 10$ m for different receiver faults for both decoupled and coupled solutions. Titles of each panel show the angle of the received fault with respect to the x-axis where positive rotation angle indices rotation towards the y-axis. Slip is always assumed to be right lateral on the receiver faults. Thus the first row with angle 0$^\circ$ represents receiver faults parallel to the x-axis, where the rupture occurs, and with the same direction of slip as the rupturing fault. While it is clear that the decoupled and the coupled cases are not identical, they do seem broadly consistent. However, the effective normal stress for $-45^\circ$ in the dynamic range (>20 m from the source) we observe opposite sign in the effective normal stress. All colours saturate at ±3 MPa to visualize all the panels with the same scale. The short black line shows the dimension of the source

**Fig. 8:**
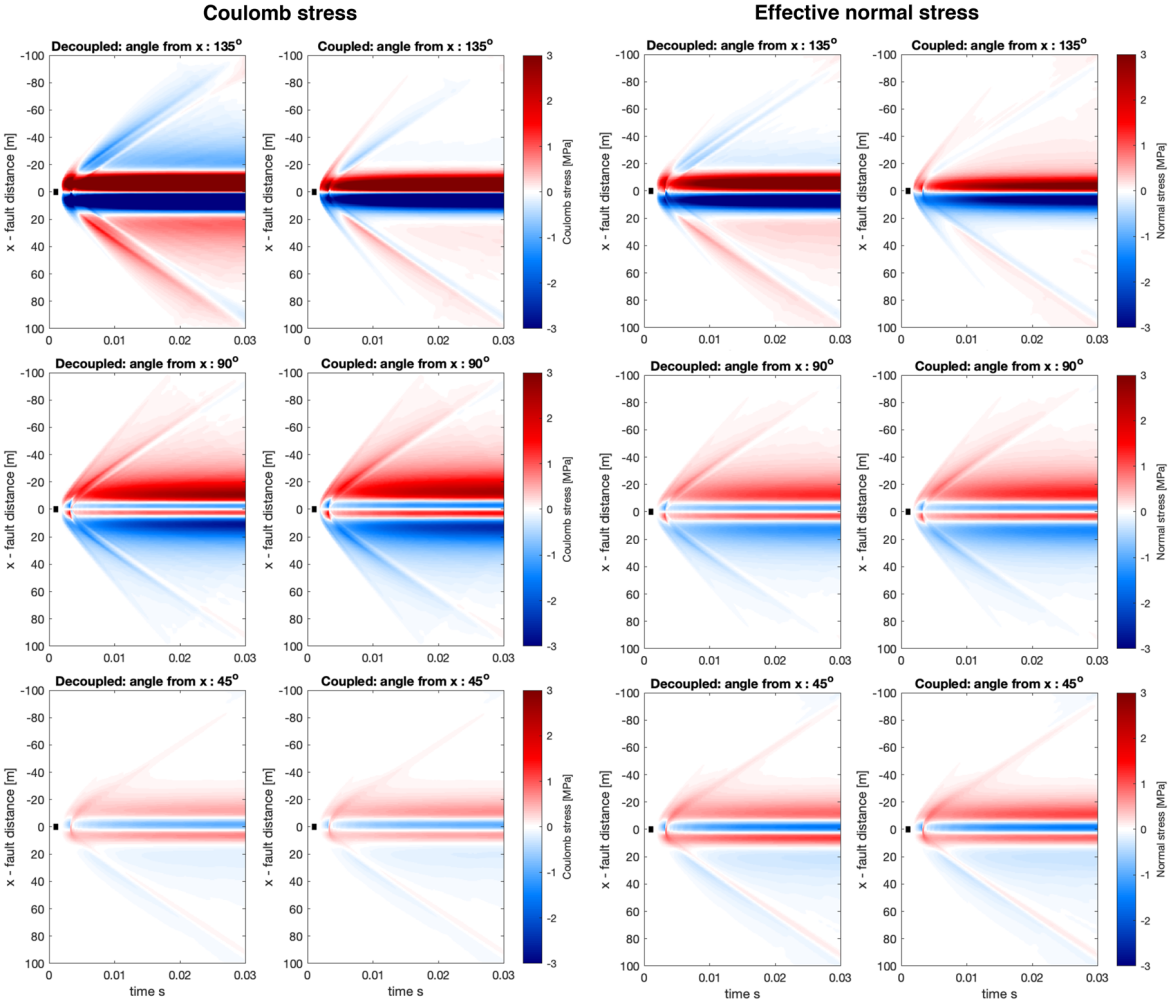
Same as Fig. 7 but showing more of the receiver fault. Here we observe substantial differences in both Coulomb stress and effective normal stress for the 135$^\circ$ angle

The Coulomb stress and effective normal stress calculations for the simple source model (see Fig. 2 for visualization of the source) has revealed several interesting phenomena. First, we observe that, on the whole, the decoupled and coupled simulations are broadly consistent. However, the largest differences are found at fault rotation of 135$^\circ$ and $-45^\circ$ angles, which correspond to the same fault plane but different sense of slip. In this case, the effective normal stress at distances exceeding about 20 m has different signs depending on if the simulation is decoupled or coupled, and this translates into differences in the Coulomb stress. Second, we observe that the onset and magnitude of the near-field quasi-static response (within 20 m distance from the fault) can be somewhat less abrupt and less intense than in the coupled compared to the decoupled simulations. For example, angles $-45^\circ$ or $-90^\circ$ in Fig. 7. Finally, we highlight the complexity of the dynamic stress interactions in Figs. 7 and 8, both in terms of magnitude, sign and spatio-temporal variability even though the source is simple and the observed stresses are in the intermediate and far-field, thus suggesting that dynamic triggering can be difficult to model and compare to field data.

**Fig. 9:**
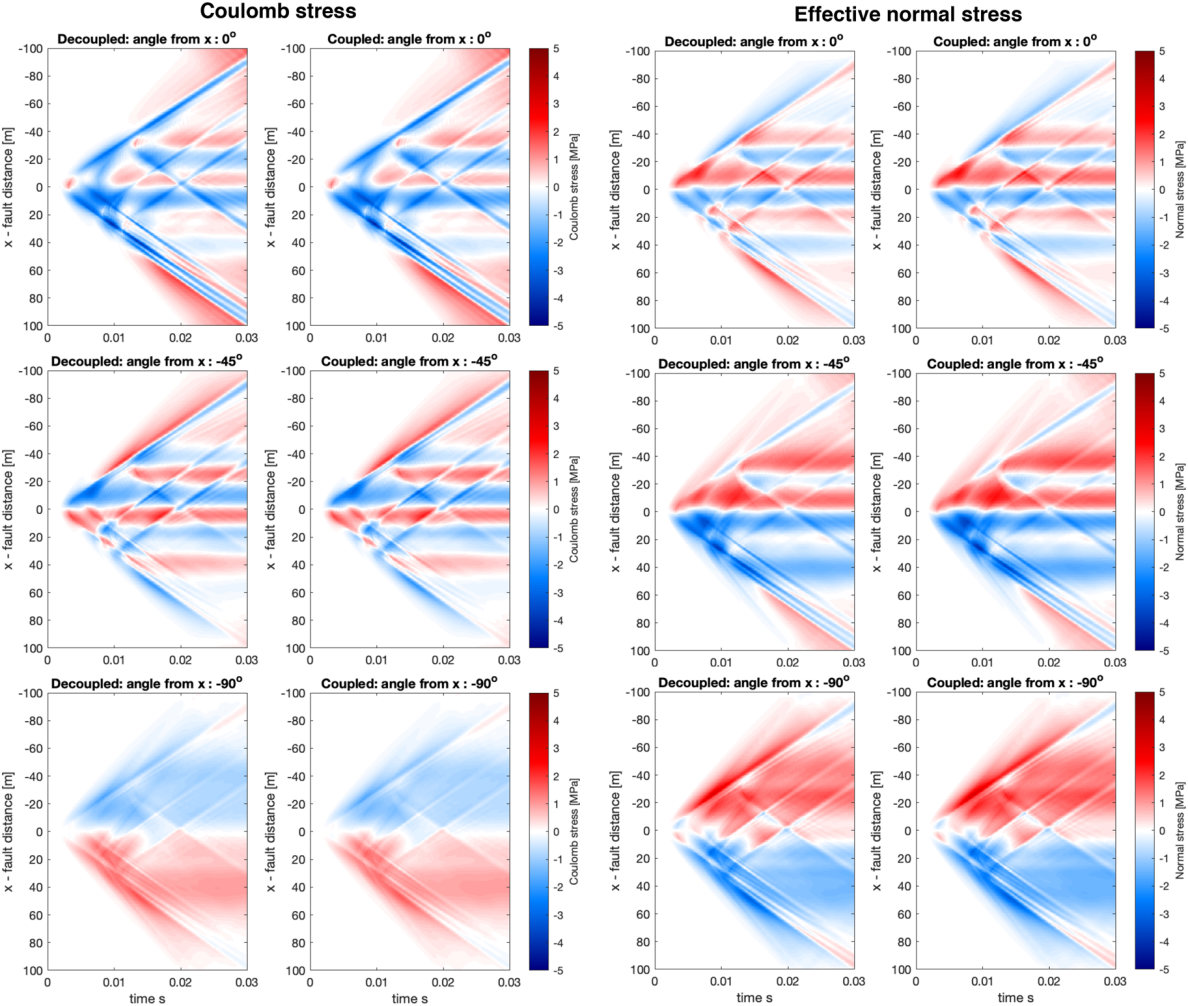
Same as Fig. 7 but for a complex near-field source

**Fig. 10:**
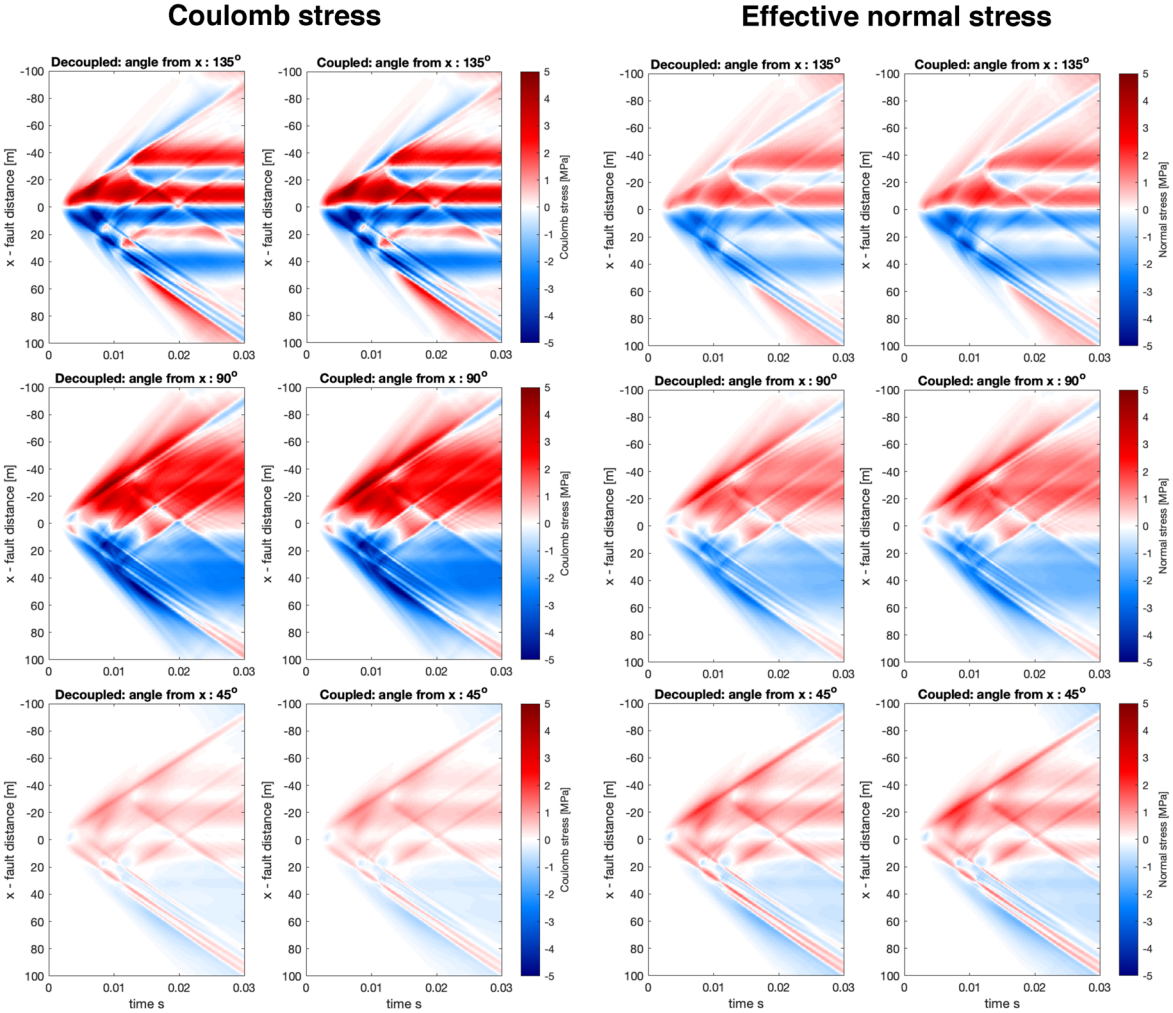
Same as Fig. 8 but for a complex near-field source

Next we’ll investigate a complex near-field source process (see description of the source in Fig. 2) with results presented in Figs. 9 and 10.

In general, we observe for the complex near-field source a remarkable agreement between the decoupled and coupled simulations (Figs. 9 and 10). In contrast to Figs. 7 and 8, where more differences are observed, in particular at fault rotation of 135$^\circ$ and $-45^\circ$, where the the sign of effective normal stress is reversed. This suggests that at the intermediate and far-field range, the full poroelastodynamic coupling may be more important. This may be due to the dispersive and attenuating properties of the poroelastodynamic medium. The complex source demonstrates that the stress interaction at this distance range can be very complex. Even for a parallel fault with the same slip direction (Fig. 9, angle 0$^\circ$), there is not a complete stress shadow effect adjacent to the source region. This is primarily due to complexities in the slip distribution and considering the effect of pore-pressure in the effective normal stress, which is then used to compute the Coulomb stress. We have thus demonstrated a type of heterogeneity, alongside others (e.g. Smith and Dieterich 2010), can explain the presence of aftershocks adjacent to fault planes in a region of a stress shadow in a smoother and less heterogeneous model.

## Discussion

### Simulating longer time

In this paper, we focused our attention on the short-term dynamic response and, in fact, only investigate a time window of 0.03 s. However, the dynamics of a poroelastic solid are not only influenced at the time-scale of wave propagation but also at the time-scale of diffusion. Our approach could, of course, be extended over a longer time and thus accounting for deformation on the diffusional time-scale simply by extending the time scale over which the kernel is evaluated. However, some care needs to be taken. First, for each wavenumber *k* the diffusional time-scale should be well temporally resolved. The diffusional time-scale in the bulk is $1/(k^2 c)$ (Heimisson et al. 2021), where *c* is the hydraulic diffusivity. We thus observe a very strong dependence on the wavenumber, and the maximum time-step in discretizing the kernel should reflect that. Another important aspect of simulating a longer time-scale is the periodic boundary conditions imposed by the spectral boundary integral approach. Thus waves do not leave the domain if traveling parallel to the x-axis. For this, there may be two solutions, first, truncation of the convolution kernels at a certain time similar to Lapusta et al. (2000). In this case, we postulate that one needs to separate the kernel into a dynamic part and a quasi-static part and only truncate the dynamic part. How to implement this part requires further investigation. Secondly, one would need to adapt the approach of Cochard and Rice (1997); Noda (2021), but this is likely not trivial.

### Extension to 3D

The method presented can also be applied to 3D problems. This would require taking a 2D Fourier transform in Eqs. 1 and 2, but otherwise follow nearly identical steps. The main issue is that to obtain the same spatial resolution as for plane strain simulations with *n* Fourier modes, one needs $n^2$ Fourier modes, which may require substantial computational resources. However, due to the fully parallel nature of the kernel computation, this can be done in theory relatively fast if the resources are available.

### Wider applicability

The general method we have presented to construct the spectral convolution kernels and using a numerical inversion of the Laplace transform could be applied more widely to obtain spectral boundary integral solutions for problems that cannot be solved fully analytically. For example, the method could be extended to problems with a more complex bulk, for example, with fault parallel layered structure or more complex properties such as thermo-poroelastic. The approach can also compliment new numerical strategies that couple spectral boundary integrals with finite elements for effeciency and wave absorption (Ma et al. 2019) and the desired boundary conditions for finite element domain can be tailored without much analysis by hand.

## Conclusions

Here we have presented a spectral boundary integral approach to simulate, understand, and analyze finite fault slip and earthquake ruptures in a poroelastodynamic solid. Our analysis and focus have been on plane strain ruptures, but a comparable approach could be applied to a 3D problem. The methodology is based on numerically constructing a convolution kernel. Once the convolution kernel has been constructed, the simulation of dynamic and static fields can be carried out very efficiently. The first step of constructing the kernel is computationally expensive but trivially parallelizable such that the only significant limit on computational time is the computation resources available. The second step, which is the actual convolution, is computationally efficient. Since the boundary integral method does not easily lend itself to account for the heterogeneity of the bulk, we suggest that this approach is most promising to simulate the bulk response at distances comparable to the fault rupture size.

With this new method, we investigate two problems. First, we try to address a practical issue by experimenting if we can solve the corresponding elastic problem and use an undrained pore-pressure response (decoupled) to simulate the problem. We find that for a complex and near-field seismic source, the agreement between the decoupled approached and the fully coupled poroelastodynamic approach is remarkably good. However, for a simple source at intermediate to far-field distances, there are some significant differences observed, in particular in the effective normal stress on receiver faults. We suggest that this is caused by the dispersive and attenuating effects introduced by the full poroelastodynamic solution. Second, we investigate the dynamic pore-pressure response. We highlight that the P-wave carries a significant pore-pressure change over large distances. P-wave arrival is associated with a peak in pressure, but the pressure then decreases again and may or may not recover later on, depending on if the observation point is close enough to be affected by the quasi-static response. We suggest that high-rate pressure measurements near-fault may offer significant insight into source processes.
